# Supplementary material for: Long Non-Coding RNAs in Drug Resistance of Gastric Cancer: Complex Mechanisms and Potential Clinical Applications
Source: Biomolecules. 2024 May 22;14(6):608. doi: 10.3390/biom14060608 (PMC11201466; doi:10.3390/biom14060608)
Supplement: Supplementary file 1 [file biomolecules-14-00608-s001.zip › biomolecules-2933045-supplementary.pdf]

Supplementary Table S1. Mechanisms and effects of potential clinical targets in drug resistance progress.

LncRNAs	Application	Ref
**oe-lncRNAs**		
ADAMTS9-AS2	increasing the sensitivity of CDDP,inhibiting proliferation	108
CRNDE	increasing the sensitivity of druy,inhibiting autophagy	121
GAS5	increasing the sensitivity of Adriamycin	107
KLF3-AS1	increasing the sensitivity of CDDP,inhibiting proliferation,metastasis	40
LEIGC	increasing the sensitivity of 5-Fu,inhibiting EMT	59
**si-lncRNA**		
AK022798	inhibiting DR,promoting apoptosis	114
ARHGAP5-AS	inhibiting MDR,proliferation,metastasis,promoting apoptosis	103
ANRIL	inhibiting MDR,proliferation,metastasis,promoting apoptosis	47
BLACAT1	inhibiting DR,metastasis,promoting apoptosis	61
DUSP5P1	inhibiting DR,proliferation,metastasis	62
DANCR	inhibiting DR,proliferation,promoting apoptosis	89
EIF3J-DT	inhibiting MDR,autophagy,promoting apoptosis	120
FEZF1-AS1	inhibiting MDR,proliferation,autophagy	104
FAM84B-AS	inhibiting DR,proliferation,promoting apoptosis	39
FGD5-AS1	inhibiting DR,proliferation	94
HOTAIR	inhibiting DR,proliferation,promoting apoptosis	36
	inhibiting DR,proliferation,promoting apoptosis	37
HOTAIR	inhibiting DR,proliferation,promoting apoptosis	116
HULC	inhibiting DR,promoting apoptosis	87
	inhibiting DR,autophagy,promoting apoptosis	88
HMGA1P4	inhibiting DR,proliferation,promoting apoptosis	90
HCP5	inhibiting MDR,stemness,proliferation,promoting apoptosis	76
	inhibiting DR,proliferation,promoting apoptosis	77
	inhibiting DR,proliferation	78
HAGLR	inhibiting DR,proliferation	95
LINC01572	inhibiting DR,autophagy,promoting apoptosis	125
LINC00922	inhibiting DR,proliferation,metastasis,promoting apoptosis	43
MRUL	inhibiting MDR,proliferation,promoting apoptosis	96
MALAT1	inhibiting DR,autophagy,promoting apoptosis	50
	inhibiting DR,proliferation,EMT,promoting apoptosis	52
	inhibiting DR,autophagy,promoting apoptosis	54
MACC1-AS1	inhibiting DR,stemness,promoting apoptosis	75
PITPNA-AS1	inhibiting DR,proliferation,apoptosis	93
SNHG6	inhibiting DR,proliferation,metastasis,promoting apoptosis	41
SNHG5	inhibiting DR,promoting apoptosis	117
SUMO1P3	inhibiting MDR,proliferation,metastasis	56
SNHG1	inhibiting DR,metastasis	95
ST7-AS1	inhibiting DR,proliferation,metastasis,promoting apoptosis	41
UCA1	inhibiting MDR,promoting apoptosis	98
	inhibiting DR and growth,promoting apoptosis	99
	inhibiting DR,promoting apoptosis	100

PVT1	inhibiting MDR,growthmetastasis,promoting apoptosis	44-46
UCA1	inhibiting DR,growth	97
**sh-lncRNA**		
ABL	inhibiting MDR,metastasis	118
ASB16-AS1	inhibiting DR,stemness,proliferation	91
ANRIL	inhibiting DR,proliferation,promoting apoptosis	48
BCAR4	inhibiting DR,stemness,promoting apoptosis	72
CRNDE	inhibiting DR,autophagy	122
CBSLR	inhibiting DR,ferroptosis	128
CRART16	inhibiting DR,proliferation	106
D63785	inhibiting DR,proliferation,metastasis,promoting apoptosis	57
FOXD1-AS1	inhibiting DR,proliferation	92
H19	inhibiting DR,proliferation,EMT,promoting apoptosis	83
HNF1A-AS1	inhibiting DR,proliferation,metastasis	60
LINC-PINT	inhibiting DR,proliferation,metastasis,promoting autophagy	42
LINC00942	inhibiting DR,stemness,promoting apoptosis	74
MALAT1	inhibiting MDR,autophagy,promoting apoptosis	49
	inhibiting DR/RR,stemness,promoting apoptosis	51
	inhibiting DR,autophagy,promoting apoptosis	53
MVIH	inhibiting DR,proliferation,metastasis,promoting apoptosis	70
NEAT1	inhibiting DR,proliferation,metastasis,promoting apoptosis	69
NUTM2A-AS1	inhibiting DR,proliferation,metastasis	68
SNHG12	inhibiting MDR,proliferation	105
SLCO1C1	inhibiting DR,proliferation,metastasis	63
THOR	inhibiting DR,stemness,promoting apoptosis	73
ZFAS1	inhibiting MDR,proliferation,metastasis,promoting apoptosis	55
**CRISPR/Cas9-lncRNA**		
PANDAR	inhibiting MDR,proliferation,promoting apoptosis	102
**exo-si-lncRNA**		
HOTTIP	inhibiting DR,proliferation,metastasis,promoting apoptosis	38
FGD5-AS1	inhibiting DR	135

Abbreviations: MDR: Multiple drug resistance; DR: Drug resistance; EMT: Epithelial-Mesenchymal Transition
oe-lncRNA: The target gene was up-regulated by transfection of overexpression vector
si-lncRNA: The target genes were down-regulated by small interfering RNAs
sh-lncRNA: The target genes were down-regulated by transfection of short hairpin vector
CRISPR/Cas9-lncRNA: The target genes were down-regulated by transfection of CRISPR /Cas9 system
exo-si-lncRNA: The target genes were down-regulated by small interfering RNAs in exosomes
